# Utility of Pretreatment Bilirubin Level and *UGT1A1* Polymorphisms in Multivariate Predictive Models of Neutropenia Associated with Irinotecan Treatment in Previously Untreated Patients with Colorectal Cancer

**DOI:** 10.1111/j.1753-5174.2008.00014.x

**Published:** 2008-12

**Authors:** Luis Parodi, Eve Pickering, Laura A Cisar, Doug Lee, Raoudha Soufi-Mahjoubi

**Affiliations:** * Pfizer IncNew York, NY, USA; † Pfizer IncGroton, CT, USA

**Keywords:** Bilirubin, Colorectal Cancer, Irinotecan, Neutropenia

## Abstract

**Purpose:**

Statistical models for predicting hematologic toxicity were evaluated based on *UGT1A1* polymorphisms and baseline serum bilirubin.

**Methods:**

Blood DNA samples were collected from 113 patients with untreated metastatic colorectal cancer receiving irinotecan (FOLFIRI, n = 36; mIFL, n = 41; CapeIRI, n = 36). The primary endpoint was absolute neutrophil count nadir during first treatment cycle. Linear regression models, with increased R^2^ implying important additional predictive power, sequentially added age, sex, baseline bilirubin level, and *UGT1A1* genotype.

**Results:**

All models demonstrated low R^2^, suggesting unaccounted variables. *UGT1A1* genotype added ∼8–9% during cycle 1 and from ∼7% [mIFL regimen] to 26% [CapeIRI regimen] after cycle 1. Correlation between genotype and overall ANC nadir without regard to treatment was low (R = −0.201, *P* = 0.035). Patients with genotype 7/7 may have increased risk for severe neutropenia, but data are insufficient to characterize this. Contribution of baseline bilirubin level was negligible.

**Conclusions:**

Ability of *UGT1A1* or baseline bilirubin to predict neutropenia is low and depends on regimen.

## Introduction

Chemotherapy in conjunction with selected targeted agents has largely been responsible for steady improvements in patient survival[1], and, in patients with metastatic disease, treatment with combinations of the most active cytotoxic agents yields median overall survival in excess of 20 months[2–8]. The attainment of palliative benefit may be thwarted, however, by the unwelcome development of treatment-emergent toxicity associated with chemotherapy, which may require dose modification, interruption of therapy, or treatment discontinuation.

Techniques to prospectively identify individuals who may be at risk for development of treatment-related toxicity include clinical risk scores, assays for biologic substances with possible predictive value, and, increasingly, pharmacogenetic testing for specific polymorphisms that inform host interactions with drugs[9–13]. The toxicity of chemotherapy, as well as its efficacy, may in part be due to heritable genetic factors modulating drug activation, metabolism, clearance, and excretion that play a role in cellular and tissue responses to treatment. Predictive genetic markers could therefore be useful in selecting patients most likely to benefit from therapy or to determine optimal patient-specific treatment regimens.

**Figure 1:**
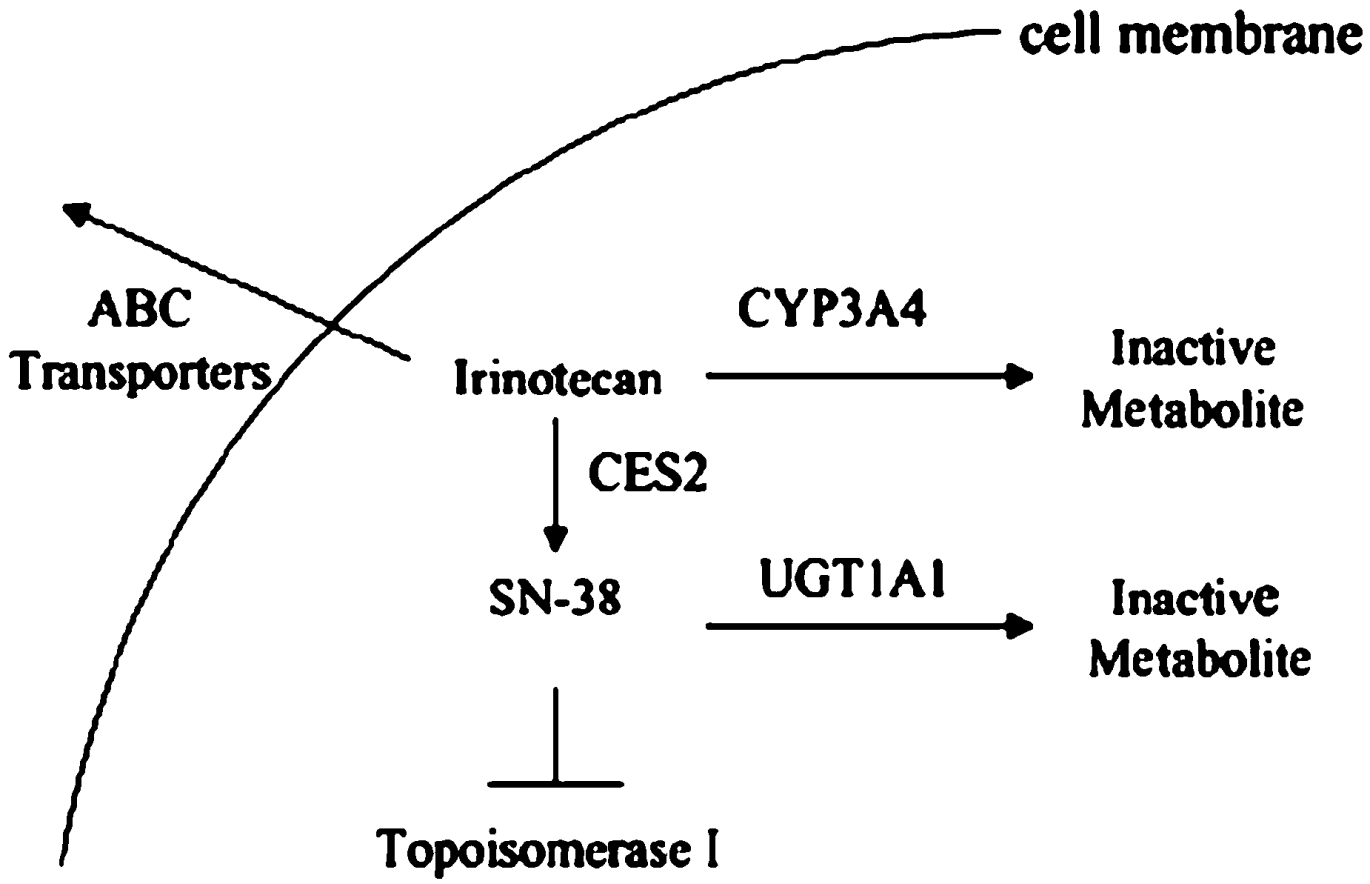
Major pathways of irinotecan metabolism and disposition. A reaction catalyzed by carboxylesterase-2 yields SN-38, the active metabolite. Glucuronidation of SN-38 to SN-38G is catalyzed by the enzyme *UGT1A1*. Several *UGT1A1* polymorphisms exist, coding for a spectrum of enzyme expression and varying ability to metabolize SN-38. This figure was published in *Semin Oncol*, 32, Tan BR, McLeod HL, Pharmacogenetic influences on treatment response and toxicity in colorectal cancer, 113–9, Copyright Elsevier (2005).

The marked interpatient variability in toxicity reported in patients with metastatic colorectal cancer (mCRC) receiving combination therapy with irinotecan, leucovorin, and 5-fluorouracil infusion (FOLFIRI) has been attributed to differences in levels of SN-38, the active metabolite of irinotecan[14]. The complex metabolism of irinotecan (Figure 1) includes inactivation of SN-38 by glucuronidation, a sequence of events mediated by the enzyme uridine diphosphate glucuronosyltransferase (UGT) 1A1[15,16]. *UGT1A1* also catalyzes the glucuronidation of bilirubin; reduced expression of UGT has been associated with disorders of bilirubin homeostasis[17–19]. Of great interest is a polymorphism in the promoter region of the *UGT1A1* gene where a variable number of repeating TA units is observed in the general population. A 6-repeat allele is the most commonly identified (wild type) form; a 7-repeat allele (designated UGT1A1*28) is associated with dramatically reduced expression of the inactivating enzyme and thus with prolonged persistence of active SN-38. The impaired ability to inactivate SN-38 in some individuals may lead to an increased risk of irinotecan-related toxicity, specifically, neutropenia[20–24].

Since *UGT1A1* plays a central role in the chemical modification of both bilirubin and the active metabolite of irinotecan, it has been suggested that pretreatment serum bilirubin levels in cancer patients reflect underlying *UGT1A1* polymorphisms and thus serum bilirubin may substitute for *UGT1A1* genotyping to risk-stratify patients for the occurrence of irinotecan-related toxicity (e.g., severe neutropenia) [21]. In this study, we assessed the contribution of baseline bilirubin level to statistical models for predicting neutropenia based on age, gender, and *UGT1A1* genotype among patients receiving first-line irinotecan-based chemotherapy for mCRC.

## Patients and Methods

### Study Design

This study was conducted as a companion study to two clinical trials evaluating irinotecan in combination with other agents in patients with mCRC. These trials included a phase III, multicenter, randomized investigation of the efficacy and safety of three irinotecan regimens (FOLFIRI, mIFL, and CapeIRI) in chemotherapy-naïve patients (BICC-C)[25] and a two-arm phase II study of irinotecan and 5-FU/LV administered with or without thalidomide[26]. All patients from whom samples were obtained for pharmacogenetic analysis signed a separate informed consent for participation in the companion study. The final protocol, any amendments, and informed consent documentation were reviewed and approved by the institutional review boards and/or Independent Ethics Committees at each of the centers participating in the study.

Participants in the BICC-C trial (N = 430) were randomly assigned to receive irinotecan 180 mg/m^2^, LV 400 mg/m^2^, 5-FU bolus 400 mg/m^2^, and infusional 5-FU 2,400 mg/m^2^ over 46 hours every 2 weeks (FOLFIRI); irinotecan 125 mg/m^2^, LV 20 mg/m^2^, and bolus 5-FU 500 mg/m^2^ weekly for 2 weeks followed by a week of no chemotherapy (modified [m]IFL); or irinotecan 250 mg/m^2^ on day 1 and capecitabine 1,000 mg/m^2^ orally twice daily for 14 days, every 3 weeks (CapeIRI). Patients underwent an additional randomization to concurrent celecoxib (400 mg orally twice daily) or placebo. Patients in the phase II study were randomly assigned to receive mIFL (as per the regimen in BICC-C) (N = 40) with or without thalidomide in a 3-week cycle. Only patients who did not receive thalidomide were included in this analysis. Of 113 samples analyzed, 107 were from the BICC-C trial and 6 were from the phase II trial.

Study participants provided separate written informed consent for genetic testing, in addition to consent obtained at entry into treatment protocols. Participation in this study was voluntary and had no bearing on participation in treatment protocols.

### Testing of Clinical Specimens

Blood was obtained (∼20 mL) from each patient for DNA extraction. All DNA extraction and genotyping was performed at a central laboratory. Identification of the *UGT1A1* promoter and determination of the number of TA repeats was performed using a high-throughput genotyping assay as described in detail elsewhere[27]. The promoter sequence with TA repeats is referred to as TA indel (insertion/deletion). Only individuals with genotypes 6/6, 6/7, and 7/7 were included in this analysis. Blood samples for bilirubin measurement were collected at participating sites during clinic visits as specified by trial protocols. Serum bilirubin and hematologic indices were assessed using standard laboratory methods.

### Statistical Methods

The analysis data set consisted of all patients with an evaluable DNA sample who received at least one dose of irinotecan. The primary outcome measurement was nadir in absolute neutrophil count (ANC) and most severe neutropenia grade during the first treatment cycle for each regimen (mIFL, FOLFIRI, and CapeIRI), with grade 3 neutropenia defined as ANC nadir below 1,000; grade 4, as ANC nadir below 500. Secondary safety endpoints were ANC nadir and most severe neutropenia grade after the first treatment cycle and during the entire treatment period. Covariates were *UGT1A1* genotype, baseline bilirubin level (continuous variable), age (continuous variable), and gender. Statistical analysis modeled the relative contributions of covariates on ANC nadir associated with a specific treatment. Due to the relatively small number of patients analyzed for each treatment, and the resulting wide confidence intervals, it was difficult to demonstrate sufficient homogeneity of the effects across treatments to allow for a pooled analysis. Therefore, a pooled analysis is not presented.

Allele and genotypic frequencies were calculated and tested for association using chi-square tests. Linear regression was used to assess the relative predictive power of the covariates for ANC nadir. Models were generated that adjusted for age and gender (model 1); age, gender, and baseline bilirubin level (model 2); age, gender, and genotype (model 3); and all 4 covariates (model 4). Partial correlation coefficients (R^2^) were used to partition variability in the primary outcome measure into relative components attributable to each covariate. The R^2^ for a model reflects the proportion of variation in response that is explained by factors included in the model. When comparing models, the difference in R^2^ provides the additional proportion of response variation that is explained by adding factors to the model; a substantial increase in R^2^ implies that the added factor contributes predictive power. Confidence intervals for the R^2^ values associated with each model were calculated with a resampling bootstrap method; if the lower limit exceeded 0 in conjunction with a substantial increase in R^2^, the additional factors in the model were considered to carry statistically significant predictive power.

## Results

### Patient Characteristics, *UGT1A1* Genotypes, and Treatment Tolerance

Blood DNA was available for 113 patients, representing 107 of 430 patients from the BICC-C study and 6 of 40 patients from the phase II study of mIFL with or without thalidomide. Of these 113 patients, 36 received FOLFIRI, 41 received mIFL, and 36 received CapeIRI. Frequencies of *UGT1A1* genotypes were approximately 44% for 6/6, 44% for 6/7, and 10% for 7/7 in the entire population (Table 1). TA indel genotypes 5/7, 5/8, and 7/8 each appeared in 1 patient. These patients were not included in the analysis. Sex, age, and performance status were comparable across treatment groups (Table 2). Median treatment exposures were 28.1 weeks (range, 2.1–105.9 weeks), 30.1 weeks (range, 1.1–95.1 weeks), and 18.1 weeks (range, 3.1–77.1 weeks) in FOLFIRI, mIFL, and CapeIRI groups, respectively.

**Table 1:** *UGT1A1* genotype of evaluable population

*UGT1A1* genotype	FOLFIRI (N = 36) n (%)	mIFL (N = 41) n (%)	CapeIRI (N = 36) n (%)
TA indel			
6/6	19 (52.8)	13 (31.7)	17 (47.2)
6/7	14 (38.9)	24 (58.5)	12 (33.3)
7/7	2 (5.6)	4 (9.8)	5 (13.9)
Other*	1 (2.8)	0	2 (5.6)

* Other represents a 5/7 patient in the FOLFIRI arm and a 6/8 and a 7/8 patient in the CapeIRI arm. These patients were not included in the model.

**Table 2:** Demographic characteristics of evaluable population

	FOLFIRI (N = 36)	mIFL (N = 41)	CapeIRI (N = 36)
Sex, n (%)			
Male	23 (63.9)	18 (43.9)	18 (50)
Female	13 (36.1)	23 (56.1)	18 (50)
Age (years)			
Mean (SD)	57.3 (10.4)	61.1 (9.8)	62.0 (12.4)
Median (min–max)	56 (37–75)	61 (41–78)	62 (26–85)
EGOG PS, n (%)			
0	19 (52.8)	22 (53.7)	21 (58.3)
1	16 (44.4)	19 (46.3)	15 (41.7)
2	1 (2.8)	0	0

*SD* = standard deviation; *EGOG PS* = Eastern Cooperative Oncology Group performance status.

Grade 4 neutropenia was experienced by patients across all treatment arms over the length of the study (2/36 patients administered FOLFIRI, 5/41 patients administered mIFL, and 4/36 patients receiving CapeIRI). First-cycle grade 4 neutropenia was experienced by one patient receiving FOLFIRI, four receiving mIFL, and three receiving CapeIRI. The most severe neutropenia grade during cycle 1, after cycle 1, and over all cycles is summarized by *UGT1A1* TA indel genotype and chemotherapy arm (Table 3).

**Table 3:** Incidence of neutropenia during cycle 1, after cycle 1, and over all cycles, by grade, genotype, and chemotherapy arm

		During cycle 1				After cycle 1				All cycles			
		Grade				Grade				Grade			
Treatment group	Genotype	1–4 n (%)	3 n (%)	4 n (%)	3 + 4 n (%)	1–4 n (%)	3 n (%)	4 n (%)	3 + 4 n (%)	1–4 n (%)	3 n (%)	4 n (%)	3 + 4 n (%)
FOLFIRI	6/6 (N = 19)	6 (31.6)	1 (5.3)	0	1 (5.3)	15 (78.9)	7 (36.8)	0	7 (36.8)	16 (84.2)	7 (36.8)	0	7 (36.8)
(Arm A)	6/7 (N = 14)	3 (21.4)	0	0	0	12 (85.7)	7 (50.0)	1 (7.1)	8 (57.1)	12 (85.7)	7 (50.0)	1 (7.1)	8 (57.1)
	7/7 (N = 2)	2 (100)	0	1 (50.0)	1 (50.0)	2 (100)	1 (50.0)	0	1 (50.0)	2 (100)	1 (50.0)	1 (50.0)	2 (100)
mIFL	6/6 (N = 13)	4 (30.8)	1 (7.7)	0	1 (7.7)	8 (61.5)	3 (23.1)	1 (7.7)	4 (30.8)	10 (76.9)	4 (30.8)	1 (7.7)	5 (38.5)
(Arm B)	6/7 (N = 24)	16 (66.7)	4 (16.7)	2 (8.3)	6 (25.0)	20 (83.3)	3 (12.5)	1 (4.2)	4 (16.7)	22 (91.7)	7 (29.2)	2 (8.3)	9 (37.5)
	7/7 (N = 4)	3 (75.0)	0	2 (50.0)	2 (50.0)	3 (75.0)	1 (25.0)	0	1 (25.0)	4 (100)	1 (25.0)	2 (50.0)	3 (75.0)
CapeIRI	6/6 (N = 17)	7 (41.2)	0	1 (5.9)	1 (5.9)	11 (64.7)	0	0	0	14 (82.4)	0	1 (5.9)	1 (5.9)
(Arm C)	6/7 (N = 12)	6 (50.0)	1 (8.3)	0	1 (8.3)	6 (50.0)	2 (16.7)	0	2 (16.7)	9 (75.0)	3 (25.0)	0	3 (25.0)
	7/7 (N = 5)	4 (80.0)	0	2 (40.0)	2 (40.0)	5 (100)	2 (40.0)	2 (40.0)	4 (80.0)	5 (100)	1 (20.0)	3 (60.0)	4 (80.0)

### Relationship of *UGT1A1* Genotype to Baseline Bilirubin Level and Toxicity

**Figure 2:**
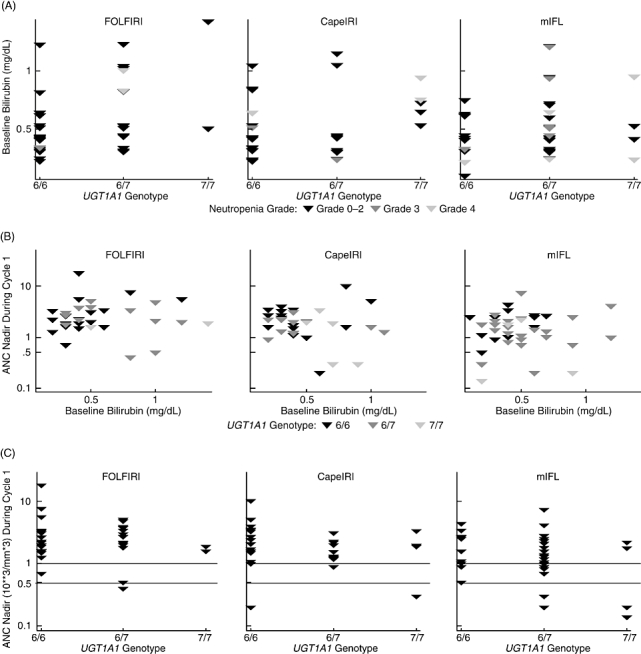
Baseline bilirubin levels and *UGT1A1* indel genotype. *A,* baseline bilirubin and absolute neutrophil count (*ANC*) nadir. *B,* ANC nadir by *UGT1A1* indel genotype. *C,* treatment group in patients receiving irinotecan.

Figure 2 shows baseline bilirubin levels and *UGT1A1* TA indel genotype by treatment group, baseline bilirubin and ANC nadir by treatment group, and ANC nadir by *UGT1A1* TA indel genotype and treatment group. Pretreatment bilirubin levels and ANC nadir did not correlate with genotype. Grade 4 toxicity occurred infrequently but was recorded among patients in all treatment groups, across all genotypes, and in all instances at normal levels of total bilirubin. Neutropenia grades 1–4 was found in every treatment group, and all grades occurred in patients with levels of total bilirubin in the normal range; high-grade hematologic toxicity most commonly occurred in patients with bilirubin levels of 1.0 mg/dL or less. The correlation between baseline bilirubin and overall ANC nadir without regard to treatment was low (R = −0.055); the correlation between genotype (number of copies of the 7 allele) and overall ANC nadir without regard to treatment was also low (R = −0.201, *P* = 0.035).

When patients were examined according to specific chemotherapy regimen, occurrences of grade 3 or 4 neutropenia were noted in small numbers of patients in all treatment groups, most commonly among patients receiving mIFL, among whom it was relatively evenly distributed across 6/6, 6/7, and 7/7 genotypes (Table 3). There was evidence of a trend indicating that patients with the 7/7 genotype have an increased risk for grade 4 neutropenia, but there were insufficient data to characterize this risk by regimen, and the heterogeneity between regimens does not justify pooling of the data for further analysis. The observed trend is similar to the association between grade 4 neutropenia and the 7/7 genotype observed by others[14,^21^,^28^,29]. One patient receiving FOLFIRI experienced first-cycle grade 3 and two had first-cycle grade 4 neutropenia. Patients receiving mIFL accounted for the most episodes of grade 3 or 4 neutropenia (3 and 4 patients, respectively). Among patients receiving CapeIRI, two had grade 3 and three had grade 4 neutropenia. There was no apparent relationship between baseline bilirubin levels and log (ANC) during first-cycle treatment or at any subsequent point during the study.

All prediction models based on *UGT1A1* TA indel genotypes had low R^2^ values (Table 4), indicating that the percentage of total variation in ANC nadir attributable to these factors was inadequate to account for the observed effects and suggesting, therefore, the presence of additional, unidentified explanatory variables. Calculated values for the additional R^2^ for adding genotype to the full model were small, ranging from 3.4% (95% confidence interval [CI], 0.0–15.4%) to 26.0% (95% CI, 6.0–51.6%).

**Table 4:** Additional utility of *UGT1A1* TA indel genotype in predictive models for first-cycle and all cycles ANC nadir containing covariates of age, sex, and total bilirubin level

	FOLFIRI (arm A) (N = 35)			mIFL (arm B) (N = 41)			CapeIRI (arm C) (N = 34)			Combined (N = 110)		
Model specification*	During cycle 1	After cycle 1	All cycles	During cycle 1	After cycle 1	All cycles	During cycle 1	After cycle 1	All cycles	During cycle 1	After cycle 1	All cycles
Demog model R^2^, %	1.9	1.5	6.0	5.0	8.8	5.8	24.4	3.6	17.9	6.5	2.4	2.3
(95% CI)	(0.0, 3.6)	(0.0, 2.8)	(0.3, 16.3)	(0.0, 9.8)	(0.2, 22.3)	(0.2, 14.8)	(5.5, 36.9)	(0.1, 10.7)	(2.6, 49.0)	(0.7, 13.7)	(0.0, 7.8)	(0.3, 4.7)
Reduced (bilirubin) model R^2^, %	2.0	1.7	7.0	5.0	9.2	5.9	24.5	3.8	27.0	6.5	2.6	2.6
(95% CI)	(0.0, 4.0)	(0.1, 3.3)	(0.3, 16.6)	(0.1, 9.7)	(0.2, 22.2)	(0.1, 15.2)	(2.5, 36.7)	(0.0, 10.2)	(5.6, 50.3)	(0.4, 13.2)	(0.0, 7.5)	(0.2, 4.4)
Reduced (genotype) model R^2^, %	9.8	9.3	10.8	13.0	15.8	20.2	32.6	28.6	41.7	13.9	11.9	6.3
(95% CI)	(0.2, 20.7)	(0.5, 19.3)	(0.6, 19.6)	(0.5, 22.5)	(0.5, 26.3)	(0.8, 35.0)	(9.4, 46.4)	(1.2, 44.8)	(11.0, 58.4)	(3.9, 22.9)	(2.6, 18.8)	(2.8, 8.6)
Full model R^2^, %	10.7	11.9	10.8	13.3	16.0	20.4	33.6	29.9	44.1	14.8	12.2	6.3
(95% CI)	(0.6, 18.8)	(1.1, 19.7)	(2.0, 19.1)	(1.8, 19.4)	(2.3, 23.7)	(2.1, 30.6)	(7.1, 46.4)	(4.1, 41.8)	(4.8, 25.1)	(4.8, 25.1)	(4.5, 18.5)	(2.8, 8.8)
Additional R^2^ for adding genotype, %	8.8	10.2	3.4	8.3	6.8	14.6	9.1	26.0	17.1	8.4	9.6	3.7
(95% CI)	(0.2, 25.0)	(0.2, 35.9)	(0.0,15.4)	(0.0, 26.8)	(0.0, 21.2)	(0.4, 37.2)	(0.5, 25.0)	(6.0, 51.6)	(4.8, 49.2)	(2.2, 16.3)	(2.8, 19.6)	(0.6, 7.8)

* Full Model: ANC nadir = age + sex + baseline bilirubin + genotype. Reduced (Bilirubin) Model: ANC nadir = age + sex + baseline bilirubin.

Demog Model: ANC nadir = age + sex. Reduced (Genotype) Model: ANC nadir = age + sex + genotype. Additional R^2^ for adding genotype = difference in R^2^ between the full model and the reduced (bilirubin) model.

Note: In the models above, ANC nadir, baseline bilirubin, and age were treated as continuous variables, and sex and genotype were treated as categorical variables.

*ANC* = absolute neutrophil count; *R^2^* = percent of total variation in ANC nadir explained by factors in the model; *CI* = confidence interval.

Age and gender, the major demographic variables, were poorly predictive of first-cycle ANC nadir for all chemotherapy regimens, especially FOLFIRI and mIFL (R^2^ = 1.9% [95% CI, 0.0–3.6%] and 5.0% [95% CI, 0.0–9.8%], respectively) in comparison with CapeIRI (R^2^ = 24.4% [95% CI, 5.5%–36.9%]). Addition of *UGT1A1* genotype to the age and gender model increased the predictive value for first-cycle ANC nadir by a comparable small amount across all treatment groups (7.9–8.2%) as well as in the combined data set (7.4%), but these slight increases were associated with wide confidence intervals (Table 4, demog and reduced genotype models).

Baseline bilirubin was largely devoid of power to predict first-cycle ANC nadir in any model, with or without *UGT1A1* genotype. Adding baseline bilirubin alone to the demographic model increased the predictive R^2^ value negligibly for all treatment regimens (0–0.1%) and had no predictive utility in the pooled data set (0%) (Table 4, models 1 and 3).

There was a trend toward a model containing *UGT1A1* TA indel genotype, age, sex, and baseline bilirubin conferring greater R^2^ values for first-cycle ANC nadir (range, 9.8 [95% CI, 0.2–20.7%] to 32.6% [95% CI, 9.4–46.4%]) than predictive models containing age, sex, and baseline bilirubin (range, 1.7 [95% CI, 0.1–3.3] to 24.5% [95% CI, 2.5–36.7%]), suggesting that *UGT1A1* TA indel genotype may offer an additional contribution to the prediction of ANC nadir beyond baseline bilirubin level. As with other statistical findings, confidence intervals were wide. This trend was consistent across all three chemotherapy arms for first-cycle ANC nadir and was also present in calculations for ANC nadir after cycle 1 and for all cycles (Table 4). Over all cycles, the contribution of the TA indel genotype was smallest for the FOLFIRI group (3.4%, [95% CI, 0.0–15.4%]) when compared with mIFL (14.6%, [95% CI, 0.4–37.2%]) and CapeIRI (17.1% [95% CI, 4.8–49.2%]).

## Discussion

Although new chemotherapy regimens have increased survival benefits in patients with colorectal cancer, toxicity leading to dose reduction and treatment discontinuation remains an obstacle to the full realization of such benefits. Thus, there is an incentive to optimize chemotherapy regimens based on the genetic profile of an individual cancer patient. Screening before chemotherapy to identify patients at risk of experiencing serious toxicities may be useful when selecting treatment regimens, adjusting dosages, or, in some cases, rejecting ineffective drugs[30].

Several studies have demonstrated that *UGT1A1* polymorphisms are associated with an increased risk for neutropenia[14,^20^,^21^,^28^,^31^–35]. A recent review of data from 10 pharmacogenetic studies of irinotecan suggests that risk for irinotecan-induced hematologic toxicity in patients positive for *UGT1A1* 7/7 is a function of irinotecan dose[29]. The power of models containing *UGT1A1* polymorphisms, baseline bilirubin, and SN-38 AUC to predict ANC nadir has been evaluated previously in heavily pretreated patients receiving doses of irinotecan ranging from 300 to 350 mg/m^2^ every 3 weeks[20,^21^,36]. The objective of the current study was to evaluate the predictive power of statistical models that include baseline serum bilirubin level and single nucleotide polymorphisms for *UGT1A1* to predict ANC nadir in patients with mCRC receiving irinotecan in the first-line setting.

There were 113 patients in the evaluable population treated with one of three different irinotecan-based first-line therapies. Although this population was small, the numbers were sufficient for exploratory analyses within the broad confidence intervals. Among all patients combined, *UGT1A1* genotype significantly predicted the rate of grade 4 neutropenia. Moreover, for each irinotecan-based chemotherapy regimen, there was a trend suggesting that *UGT1A1* genotype contributes modestly to the prediction of ANC nadir during irinotecan treatment. This effect was similar across all treatment groups, although confidence intervals for the relationship were wide. The additional utility of adding *UGT1A1* genotype based on the TA indel was ∼8–9% during the first treatment cycle for all regimens, ranged from 7–26% after the first treatment cycle, and was between 3% and 17% across all cycles. These findings suggest a modest role, at best, for pharmacogenomic profiling in irinotecan-based therapy.

Also, in the current study, baseline bilirubin levels were not correlated with ANC nadir, and the addition of baseline bilirubin to the predictive model incorporating age, sex, and *UGT1A1* genotype failed to improve the explanatory power of the model for ANC nadir. These results suggest that there is no clinically useful relationship between bilirubin level and irinotecan hematologic toxicity in chemotherapy-naïve adults with mCRC treated with standard irinotecan-containing first-line regimens. This is in contrast to an evaluation of 86 patients in which pretreatment bilirubin level was strongly associated with the development of severe neutropenia[20,^21^,36]. In these studies, a majority of patients had been pretreated with other regimens, which may have affected their ability to metabolize SN-38, while in our investigation, all patients were chemotherapy-naïve. Patients in the other reports received high-dose irinotecan monotherapy (300–350 mg/m^2^ every 3 weeks), whereas the present study evaluated combination therapies utilizing lower doses of irinotecan, including FOLFIRI, which has become a standard front-line treatment. However, both studies confirm that *UGT1A1* testing has low sensitivity to predict severe neutropenia, as indicated by low R^2^[21,36]. This lack of predictive power limits the use of *UGT1A1* genotyping in providing treatment guidance prior to initiating therapy.

Currently, unidentified factors may significantly add to predictive accuracy for ANC nadir, arguing against reliance on *UGT1A1* genotype alone, which has little predictive utility.

With the continuing introduction of newer and more effective agents, combination therapies introduce the possibility that one drug may influence the activity of an enzyme involved in the metabolism of another[37]. Apart from genetic factors affecting the complex metabolism of irinotecan, which involves multiple enzymatic and transportational processes enacted at various cellular locales and requires participation of the cytochrome p450 system, efflux pumps, and other mechanisms[1,^38^–40], over 29 genes are implicated in the integrated metabolism of 5-FU, a core component of the FOLFOX and FOLFIRI regimens. Genetic variation in any of these genes can affect clinical response or toxicities[41,42]. Additionally, the toxicities experienced in this study may have been at least partially a result of simultaneous exposure to the multiple drugs comprising the mIFL, CapeIRI, and FOLFIRI regimens, with or without the addition of celecoxib or thalidomide, rather than to irinotecan as a single component of these complex treatment protocols. A limitation of the current study is that we did not undertake pharmacokinetic analyses of SN-38 levels either prior to or following exposure to irinotecan; variations in levels among patients may have relevance to the likelihood of increased toxicity reactions. Diarrhea also was not included as an endpoint in our study and consequently is not included in the model.

The utility of pretreatment screening for a single nucleotide polymorphism prior to the administration of sophisticated regimens employing combinations of agents remains to be determined. Polymorphisms involved in drug metabolism do not act in isolation[41]; neither is the presence of a specific, single nucleotide polymorphism an unequivocal indicator that an individual patient will show an altered response[43]. Variability in drug actions reflect heritable changes in an individual patient's metabolism of the drug, its specific target, and the complex biologic milieu in which drugs and their target molecules interact[44]. Focus on single-gene polymorphisms, e.g., 6/6, 6/7, or 7/7, may be less useful than screening for interindividual variations in multiple processes that comprise a pharmacokinetic pathway[37]. Investigations geared toward comprehensive analysis of numerous metabolic and degradative genetic components, and delineation of the functional importance of genetic variants across a range of drug pathway genes, may represent a more useful approach to integrating pharmacogenetic testing in cancer chemotherapy[41,45].

Focusing on comprehensive genetic profiles rather than on single specific polymorphisms offers the promise of enormous benefit from chemotherapy; recently, a genetic signature comprising 14 genes identified in tumors of patients with mCRC was predictive of response to FOLFIRI (100% specificity); however, this signature needs to be validated in an independent cohort of patients[46]. Technologic and computational obstacles remain before comprehensive genetic analysis is fully integrated into clinical care[44,^47^,48].

This study demonstrates that the ability of *UGT1A1* to predict neutropenia is, at best, modest. Adding baseline bilirubin to the model containing *UGT1A1* genotype does not substantially increase explanatory power. Determining the specific contribution to the development of toxicity of variations of genes involved in drug-metabolizing processes can be difficult, given patients’ heterogeneous backgrounds and the complex physiologic changes that can be caused by multiple, confounding factors, including comorbid conditions, organ dysfunction secondary to previous treatments, tumor behavior, nutritional status, and effects of concurrent medications[37,49]. It is not surprising, therefore, that a single polymorphism may not contribute sufficient information for assessment of toxicity risk beyond that suggested by clinical indicators.
